# Detection of Silver and Mercury Ions Using Naphthalimide-Based Fluorescent Probe

**DOI:** 10.3390/molecules29215196

**Published:** 2024-11-02

**Authors:** Chunwei Yu, Xiangxiang Li, Mei Yang, Yinghao Xie, Jun Zhang

**Affiliations:** NHC Key Laboratory of Control of Tropical Diseases, School of Tropical Medicine, Hainan Medical University, Haikou 571199, China; cwyu@muhn.edu.cn (C.Y.); lxx@hainmc.edu.cn (X.L.); yang24364@hainmc.edu.cn (M.Y.); sos3126444394@gmail.com (Y.X.)

**Keywords:** naphthalimide, fluorescent probe, Ag^+^, Hg^2+^, cell imaging

## Abstract

A multifunctional fluorescent probe P based on a naphthalimide derivative for the detection of Ag^+^ and Hg^2+^ through a dual-signal was designed and characterized. P exhibited a large Stokes shift (107 nm), high selectivity, good sensitivity, and fast response time. By adjusting the testing medium and the order of reagent addition, multifunctional detection with P was achieved. The addition of Ag^+^ or Hg^2+^ to P solution in either ethanol or an ethanol–water mixture resulted in a significant quenching of fluorescence emission at 537 nm and caused a decrease in the absorbance at 440 nm accompanied by the appearance of a new absorption peak at around 340 nm, and there was an obvious color change from yellow to colorless. In contrast, the addition of other common metal ions and anions did not produce substantial spectral or color changes. The detection limit of probe P for Ag^+^ and Hg^2+^ was calculated to be 0.33 μM. The sensing mechanism was proposed and validated through MS and ^1^H NMR spectrometry methods. Additionally, P demonstrated the capability to recognize Ag^+^ and Hg^2+^ in living cells with satisfactory results.

## 1. Introduction

Silver and mercury are two heavy metals that have significant adverse effects on both the environment and human health. Silver, as a precious metal, poses considerable risks to aquatic organisms even at low concentrations. When silver enters the human body through contaminated water, it can accumulate in various organs, leading to vision impairment and triggering seizures [1]. Mercury is one of the most toxic metals, capable of migrating and dispersing through air and water. It can persist in the environment for extended periods and continuously accumulate in the food chain. Mercury accumulation in the human body can cause severe health issues, such as cognitive impairment, motor disorders, blindness, and developmental abnormalities in embryos, and can be life-threatening in extreme cases [2]. Therefore, the detection of trace amounts of silver and mercury in the environment is of utmost importance.

Traditional metal ion detection techniques, such as atomic absorption spectroscopy, electrochemical methods, and inductively coupled plasma optical emission spectrometry, are widely employed for the detection of Ag^+^ and Hg^2+^ [3,4,5]. While these methods offer advantages, including rapid detection and high sensitivity, they also present several significant drawbacks, including high instrument costs, complex sample pretreatment processes, substantial solvent consumption, and the necessity for specialized operators, all of which limit their practical applications. In contrast, fluorescent probes have garnered considerable attention due to their simplicity, affordability, real-time detection capabilities, and non-destructive nature [6,7]. Particularly, the design and synthesis of straightforward yet effective small-molecule fluorescent probes for the targeted detection of metal ions have become a prominent area of research [8]. Although numerous probes for Hg^2+^ have been developed [9,10,11], reports on fluorescent probes for Ag^+^ remain relatively limited [12,13,14]. This scarcity can be attributed to the moderate coordination ability of silver ions, making it challenging to differentiate them from other heavy metals. Moreover, most existing probes are designed to detect only a single target ion. Recently, the concept of a “single probe for multiple analytes” has gained traction. This innovative strategy can be realized by distinguishing different ions through varied excitation wavelengths, different solvents, or via UV-vis and fluorescence spectroscopy [15,16,17]. The advancement of novel probes capable of detecting multiple metal ions is of great significance, as they offer enhanced efficiency compared to traditional single-target detection methods. Though multifunctional fluorogenic probes had been reported for the selective detection of metal ions like Cu^2+^ and Al^3+^ [18], Ag^+^ and Cu^2+^ [19], Cu^2+^ and Pb^2+^ [20], reported dual-site probes for the simultaneous detection of multiple metal ions are still rare. So, developing a dual-site probe for Ag^+^ and Hg^2+^ is particularly urgent.

Common fluorescent fluorophores used in these probes include quinoline, rhodamine, fluorescein, naphthalimide, and coumarin. Naphthalimide-based fluorescent probes are widely used in metal ion detection and bioimaging due to their high fluorescence quantum yields, long emission wavelength, good solubility, and biocompatibility. Additionally, their structural tunability and simple synthesis process make them important research tools [21,22]. Thiosemicarbazide motifs, characterized by their sulfur (S) and nitrogen (N) donor atoms, demonstrate a strong affinity for thiophilic or aminophilic metal ions, such as Ag^+^. In previous work, we successfully introduced amino thiourea into the naphthalimide framework through a Schiff base reaction, achieving the highly selective recognition of silver ions via C=N isomerization and the photoinduced electron transfer (PET) mechanism [23]. Furthermore, some rhodamine- and BIDIPY-based probes have utilized thiourea for the recognition of Hg^2+^ [24,25]. Inspired by these findings, this study explored the potential of incorporating the thiourea unit into the 1,8-naphthalimide structure for the selective detection of Ag^+^/Hg^2+^.

## 2. Results and Discussion

### 2.1. Selective Analysis of P to Metal Ions and Anions

**Figure 1:**
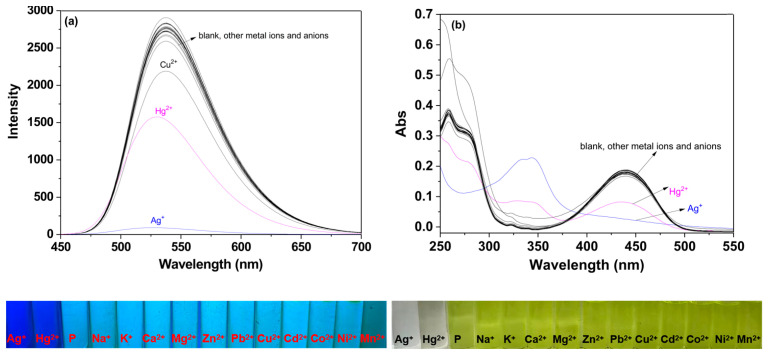
Fluorescence spectra (**a**) and ultraviolet spectra (**b**) of P (10 μM) in ethanol upon addition of various metal ions and anions (100 μM). Bottom: photograph of the solution of P in the absence and presence of the metal ions under ambient light and UV-vis light.

In the ethanol solution of P, 100 μM of various metal ions (Na^+^, K^+^, Ag^+^, Ca^2+^, Mg^2+^, Zn^2+^, Pb^2+^, Cu^2+^, Hg^2+^, Cd^2+^, Co^2+^, Ni^2+^, Mn^2+^) and anions (I^−^, NO_3_^−^, Cl^−^, Ac^−^, ClO_4_^−^, Br^−^, H_2_PO_4_^−^, HPO_4_^2−^, CO_3_^2−^, SO_4_^2−^) was added. The fluorescence and UV-vis spectra of the system were recorded (Figure 1). As shown in Figure 1a, P exhibited strong fluorescence at 537 nm. Upon the addition of Ag^+^, the fluorescence at 537 nm was completely quenched, achieving a quenching rate of 100%. Although the introduction of Hg^2+^ also led to a decrease in the fluorescence intensity of P, the quenching rate was only 47%, and a significant fluorescence emission remained. The addition of other metal ions and anions did not result in any noticeable reduction in fluorescence, indicating that P demonstrated excellent selectivity for Ag^+^ in ethanol. In Figure 1b, prior to the addition of ions, P displayed a characteristic absorption peak of naphthalimide at 440 nm. The introduction of Hg^2+^ caused a reduction in absorbance at 440 nm, along with a weak absorption observed near 340 nm. Following the addition of 100 μM of Ag^+^, the absorption peak of P at 440 nm disappeared, while a strong absorption peak emerged around 340 nm, confirming that P possessed a good selectivity for Ag^+^. Moreover, the colorimetric tubes containing blanks and various added ions all exhibited a yellow color, but only the addition of Ag^+^ caused the P solution to turn colorless, which can be attributed to the specific quenching effect of Ag^+^ on P. The photographs displaying the solution of P in both the absence and presence of metal ions under ambient light and UV-vis light are presented in Figure 1. These images effectively demonstrated the potential for practical applications in the naked-eye detection of these analytes.

**Figure 2:**
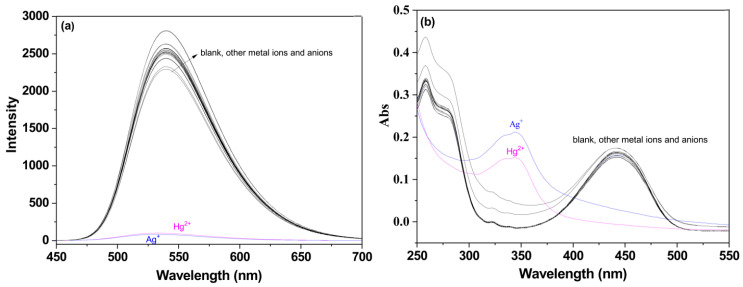
Top: fluorescence spectra (**a**) and UV-vis spectra (**b**) of probe P (10 μM) in ethanol–water solution (*v*:*v*, 9:1, pH 7.0, 20 mM HEPES) of various metal ions and anions (100 μM).

Interestingly, when the test medium was adjusted to an ethanol–water solution (*v*:*v*, 9:1, pH 7.0, 20 mM HEPES), and the order of reagent addition was changed through adding P first and then the tested ions, the fluorescence and UV-vis detection results were shown in Figure 2. As seen in Figure 2a, the addition of 100 μM Ag^+^ or Hg^2+^ resulted in a significant quenching of the fluorescence intensity of P at 537 nm, with a quenching rate approaching 100%. In contrast, under the same conditions, the addition of other metal ions and anions to the P solution did not cause any noticeable changes in the fluorescence spectrum. This indicated that P can selectively recognize both Ag^+^ and Hg^2+^. Furthermore, as illustrated in Figure 2b, only Ag^+^ and Hg^2+^ caused a substantial decrease in the absorbance of the characteristic naphthalimide peak at 440 nm, accompanied by the emergence of a new absorption band near 340 nm.

### 2.2. Competition Analysis of P for Detecting Ag^+^/Hg^2+^

To assess the influence of other metal ions on the selective recognition process of P, 50 μM of Na^+^, K^+^, Ag^+^, Ca^2+^, Mg^2+^, Zn^2+^, Pb^2+^, Cu^2+^, Hg^2+^, Cd^2+^, Co^2+^, Ni^2+^, Mn^2+^ was introduced into a 10 μM solution of P containing 10 μM of Ag^+^ in ethanol (Figure S1a), and Ag^+^ or Hg^2+^ in an ethanol–water solution (*v*:*v*, 9:1, pH 7.0, 20 mM HEPES) (Figure S1b,c) at 537 nm. Only Hg^2+^ caused a fluorescence decrease within a range of 20% to Ag^+^ in ethanol–water solution (*v*:*v*, 9:1, pH 7.0, 20 mM HEPES), while the interference from the other metal ions remained minimal, typically below 5%. These test results demonstrated that other common metal ions had little effect on the selective detection of Ag^+^ and Hg^2+^ by P, which showed high anti-interference ability for the fluorogenic sensing of Ag^+^ and Hg^2+^ in ethanol or ethanol–aqueous solution.

### 2.3. Sensitivity Analysis of P for Detecting Ag^+^/Hg^2+^

**Figure 3:**
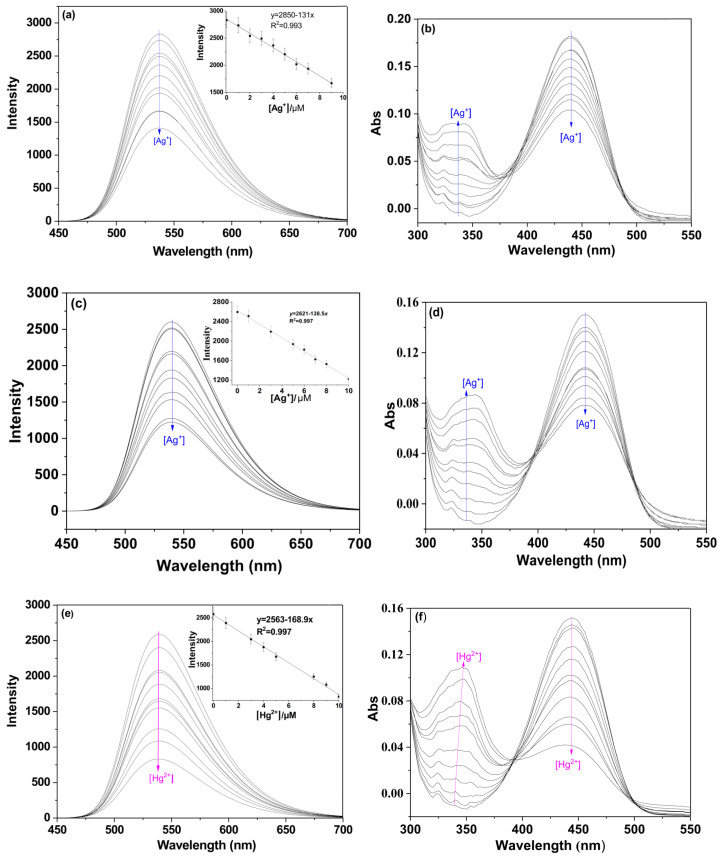
(**a**) Fluorescence spectra of P (10 μM) toward various concentration (1–10 μM) of Ag^+^ in ethanol. Inset: the fluorescence of P (10 μM) as a function of Ag^+^ concentration (1–10 μM); (**b**) UV-vis spectra of P (10 μM) toward various concentration (1–10 μM) of Ag^+^ in ethanol; (**c**) fluorescence spectra probe P (10 μM) toward various concentration (1–10 μM) of Ag^+^ in ethanol–water solution (*v*:*v*, 9:1, pH 7.0, 20 mM HEPES). Inset: the fluorescence of P (10 μM) as a function of Ag^+^ concentration (1–10 μM); (**d**) UV-vis spectra of P (10 μM) toward various concentration (1–10 μM) of Ag^+^ in ethanol–water solution (*v*:*v*, 9:1, pH 7.0, 20 mM HEPES); (**e**) fluorescence spectra probe P (10 μM) toward various concentration (1–10 μM) of Hg^2+^ in ethanol–water solution (*v*:*v*, 9:1, pH 7.0, 20 mM HEPES). Inset: the fluorescence of P (10 μM) as a function of Hg^2+^ concentration (1–10 μM); (**f**) UV-vis spectra of P (10 μM) toward various concentration (1–10 μM) of Hg^2+^ in ethanol–water solution (*v*:*v*, 9:1, pH 7.0, 20 mM HEPES).

We further performed fluorescence titration and UV-vis experiments to research the sensing ability of P to Ag^+^ (0–1 equiv.) in ethanol. The decreased fluorescence intensity at 537 nm can be easily found upon the addition of increasing amounts of Ag^+^. As shown in the inset of Figure 3a, the plots of the fluorescence intensity versus the Ag^+^ concentration showed a linear correlation in the range of 1–10 μM, and the detection limit for Ag^+^ was deduced to as low as 0.33 μM based on the linear correlation equation y = 2850 − 131x, with the correlation coefficient *R*^2^ = 0.993 (LOD = 3 *s*/*K*. Here, *s* is the standard deviation of the blank probe solution and *K* is the slope of the standard curve) [26]. Moreover, as shown in Figure 3b, the subdued absorption at 440 nm and enhancement at 340 nm with an isoelectric point at 392 nm could be observed with the increasing Ag^+^ concentrations concomitantly. As the reaction progressed, the color of the solution changed from yellow to transparent.

In an ethanol–water solution (*v*:*v*, 9:1, pH 7.0, 20 mM HEPES), fluorescence spectra of P containing different concentrations (0, 1, 2, 3, 4, 5, 6, 7, 8, 9, 10 μM) of Ag^+^ or Hg^2+^ were measured and were shown in Figure 3c,e, respectively. The spectral results revealed that the fluorescence intensity at 537 nm of the solution decreased with the increase in the Ag^+^ or Hg^2+^ concentration. The fluorescence intensity of the solution had a good linear relationship with Ag^+^ concentration (1–10 μM), and the linear correlation equation could be described as y = 2621 − 138.5x, with the correlation coefficient *R*^2^ = 0.997. The NaCl-adding experiments were conducted to examine the reversibility of the P. As clearly shown in Figure S2, the fluorescence intensity at 537 nm remained unaffected when NaCl was added to the solution containing P and Ag^+^, which indicated that P can irreversibly coordinate with Ag^+^. Similarly, the good linear relationship between the fluorescence intensity of the solution and Hg^2+^ concentration (1–10 μM) could be described by the equation y = 2563 − 168.9x, with the correlation coefficient *R*^2^ = 0.993, and the minimum limit of detection (LOD) of P for Ag^+^ and Hg^2+^ ions was calculated to be 0.33 μM, respectively. The Na_2_S-adding experiments were conducted to examine the reversibility between P and Hg^2+^ shown in Figure S3; the fluorescence intensity at 537 nm increased when Na_2_S was added to the solution containing P and Hg^2+^. In addition, the color also changed from colorless to yellow. When Hg^2+^ was added to the system again, the signal was almost completely reproduced, and the yellow solution turned to colorless. These findings indicated that P can reversibly coordinate with Hg^2+^. It was speculated that the coordination of Ag^+^ or Hg^2+^ with P triggered a reverse photo-induced electron transfer (PET) process, resulting in fluorescence quenching. As shown in Figure 3d,f, the trend observed in the UV-vis spectra with increasing concentrations of Ag^+^ or Hg^2+^ mirrored the changes observed when P detected Ag^+^ in ethanol (Figure 3b).

A comparative study between this work and previously reported probes was made (Table S1). Among these fluorescent probes for monitoring Ag^+^ and Hg^2+^, some detected Ag^+^ and Hg^2+^ through dual chromo- and fluorogenic changes [27,28,29,30], including our proposed probe P, which allowed for easy visual observation. Notably, the construction of a fluorescence-enhanced probe facilitated an improved signal-to-noise ratio and reduced background interference [27,31,32,33]. Additionally, a few probes exhibited a wide quantitation range [28,29,30,32,34] or achieved low nanomolar limits of detection (LOD) for Hg^2+^ [32]. However, most of these probes required more rigorous testing conditions, and their applicability in living cells was not investigated [28,30,31,34]. Probe P demonstrated high sensitivity for Ag^+^ and Hg^2+^ detection, a wide linear range, and a lower detection limit, making it suitable for detecting Ag^+^ and Hg^2+^ in cell imaging. However, it must be noted that the water solubility and sensitivity of the probe needed further improvement, which required the optimization of the probe structure.

### 2.4. Effect of Solvents, pH, and Time on P to Ag^+^/Hg^2+^

To investigate the impact of solvents on the optical properties of probe P, we analyzed its fluorescence and UV-vis spectra in various solvents, including methanol (MeOH), ethanol (EtOH), acetonitrile (CH_3_CN), dimethyl sulfoxide (DMSO), dimethylformamide (DMF), and tetrahydrofuran (THF) (Figure S4). The results showed that the maximum absorption wavelength and fluorescence intensity of probe P varied with different solvents. Ethanol was selected as the primary solvent due to its relatively strong fluorescence and distinct color change upon the addition of Ag^+^ and Hg^2+^. Though the fluorescence intensities of probe P in THF and CH_3_CN were much stronger than that in ethanol, the environmental friendliness of ethanol was a significant factor in its selection. To assess the applicability of probe P in aqueous solutions, we measured its fluorescence spectra in ethanol/water mixtures at various ratios (*v*:*v*), as shown in Figure S5. Notably, the fluorescence intensity of P was significantly quenched upon the addition of Ag^+^ in both ethanol and the EtOH/H_2_O (9:1, *v*:*v*) mixture, as well as with the addition of Hg^2+^. To further investigate the applicable pH range of P for the detection of Ag^+^ and Hg^2+^, fluorescence spectra of P (10 μM) in EtOH/H_2_O (9:1, *v*/*v*) at different pH levels after the addition of Ag^+^ or Hg^2+^ (100 μM) were measured, as shown in Figure S6. The results revealed that the fluorescence emission at 537 nm of P remained stable under acidic, neutral, and alkaline conditions with a pH of less than 10. However, after the addition of Hg^2+^, there was a remarkable decrease to almost complete quenching of the fluorescence intensity at 537 nm across these pH conditions, indicating that P can be effectively used for Hg^2+^ sensing within a pH range of 1 to 10. In contrast, upon the addition of Ag^+^, there was a slight increase from pH 7 to 4 due to the protonation of the tertiary amine, which displaced Ag^+^, with complete quenching occurring at pH 7.0 to 10. To accommodate the typical pH values encountered in practical detection environments, subsequent experiments were conducted at pH 7.0.

The time-dependent fluorescence intensity changes in P (10 μM) after adding 10 equiv. of Ag^+^ or Hg^2+^ were tested. As shown in Figures S7 and S8 in the fluorescence spectra, the emission band of P at 537 nm disappeared immediately within 5 min upon adding Ag^+^ or Hg^2+^ over the incubation time, the intensity was almost the same, showing that the time for the P to form a complex with Ag^+^ or Hg^2+^ was very short. After continuous illumination for 0.5 h, the fluorescence intensity of the P and P-Ag^+^ complex in ethanol and the P, P-Ag^+^, and P-Hg^2+^ complex in ethanol–water solution (*v*:*v*, 9:1, pH 7.0, 20 mM HEPES) did not show any obvious fluorescence change (Figure S19). The good photo-stability of the P-Ag^+^ and P-Hg^2+^ complex suggested that probe P was very favorable for us to detect Ag^+^ or Hg^2+^ qualitatively and quantitatively.

### 2.5. Mechanism Analysis of P for the Recognition of Ag^+^/Hg^2+^

**Scheme 1:**
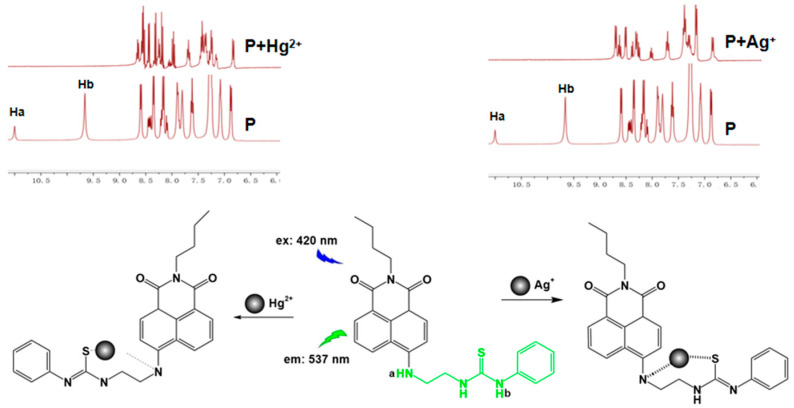
**Top**: The ^1^H NMR comparison of P with P-Ag^+^ and P-Hg^2+^. **Bottom**: Proposed recognition mechanism of P with Ag^+^ and Hg^2+^.

The recognition mechanism of P with Ag^+^ and Hg^2+^ was first studied by ^1^H NMR (Figures S10 and S11). After the addition of Ag^+^ and Hg^2+^ to the solution of P in *d_6_*-DMSO, the signals of −NH_a_ and −S=C−NH_b_ at 11.00 ppm and 9.66 ppm disappeared, which proved the participation of N and S in the formation of the P-Ag^+^ and P-Hg^2+^ complex. The binding mechanism is shown in Scheme 1.

In order to further study the reaction process, MS spectra were also conducted. The formation of a 1:1 complex was further confirmed by ESI(+)-MS analysis: an ethanol solution containing P and 1 equiv. of Ag^+^/Hg^2+^ (Figures S12 and S13) showed strong peaks at *m*/*z* 554.59 and 679.66, which belonged to [P + Ag^+^]^+^ and [P + Hg^2+^ + Cl^−^− 2H^+^], respectively. As expected, the result indicated that there was a 1:1 stoichiometry of Ag^+^/Hg^2+^ to P in the complex, which was also supported by the Benesi–Hildebrand method [35], and the association constant *K* was determined from the slope to be 5.7 × 10^4^ M^−1^ and 1.0 × 10^5^ M^−1^, respectively (Figures S14 and S15).

### 2.6. Cell Imaging

**Figure 4:**
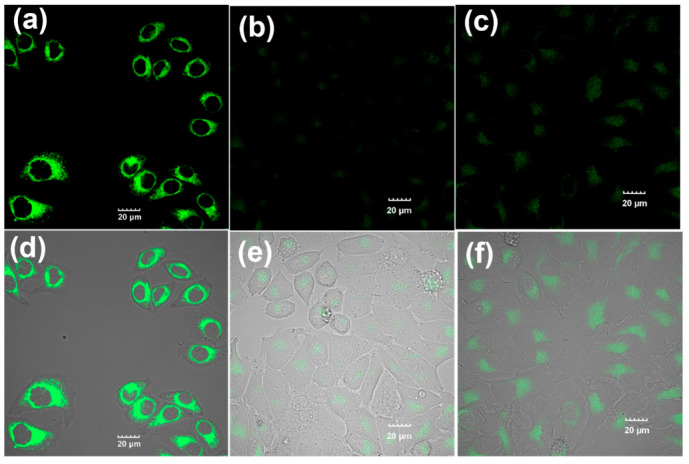
Hela cells fluorescence images. (**a**) Cells cultured with 10 µM of P for 30 min in the growth medium; (**b**) cells cultured with 10 µM of P for 30 min in the growth medium and then supplemented with 8 μM Ag^+^ for 30 min; (**c**) cells cultured with 10 µM of P for 30 min in the growth medium and then supplemented with 8 μM Hg^2+^ for 30 min; (**d**) image of bright field cells in panel (**a**); (**e**) image of bright field cells in panel (**b**); (**f**) image of bright field cells in panel (**c**).

The capacity of probe P to detect Ag^+^ and Hg^2+^ in HeLa cells was assessed (Figure 4). Upon incubation of HeLa cells with 10 μM of probe P at 37 °C for 30 min, a significantly strong fluorescence signal was detected (Figure 4a). In contrast, when these cells were subsequently treated with Ag^+^ and Hg^2+^ (8 μM) for an additional 30 min after the initial exposure to P, a notable change in fluorescence was observed (Figure 4b–c). This indicated that probe P effectively penetrated cell membranes, and the observed decrease in intracellular fluorescence was attributed to the interaction of Ag^+^ and Hg^2+^ with probe P inside the cells. Moreover, morphological evaluations of the cells suggest that probe P possessed low toxicity and good cytocompatibility. Therefore, probe P demonstrates significant promise as a tool for bioimaging Ag^+^ and Hg^2+^ in biological systems.

**Figure 5:**
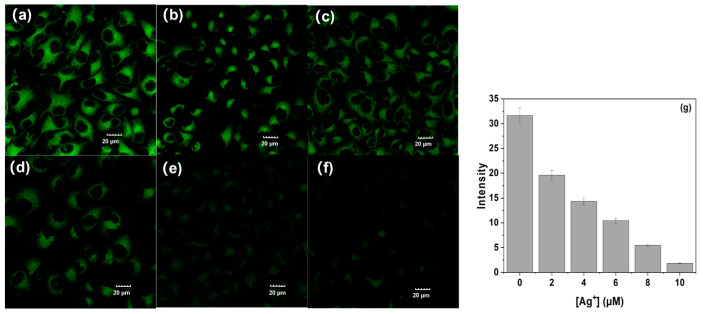
Confocal fluorescence images of Hela cells. Panels (**a**–**f**) showed cells that were incubated with 10 μM probe P in the growth medium for 30 min at 37 °C. Following this, the cells were treated with 0, 2, 4, 6, 8, and 10 μM Ag^+^ for an additional 30 min, respectively; (**g**) presented the fluorescence intensity of probe P at varying concentrations of Ag^+^ (**a**–**e**): 0, 2, 4, 6, 8, and 10 μM.

**Figure 6:**
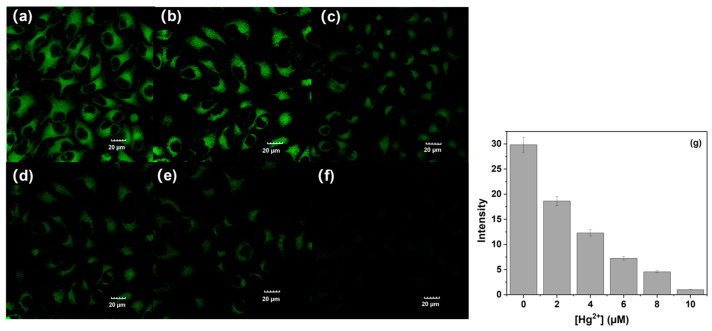
Confocal fluorescence images of Hela cells. (**a**–**f**) Cells incubated with 10 μM P in the growth media for 30 min at 37 °C and then supplemented with 0, 2, 4, 6, 8, 10 μM Hg^2+^ for 30 min, respectively; (**g**) fluorescence intensity of P with various [Hg^2+^] (**a**–**e**): 0, 2, 4, 6, 8, 10 μM.

Additionally, fluorescence imaging of probe P (10 μM) in HeLa cells was performed with varying concentrations of Ag^+^ and Hg^2+^ (0, 2, 4, 6, 8, 10 μM). The results revealed a consistent reduction in fluorescence intensity with increasing concentrations of Ag^+^ and Hg^2+^, as shown in Figure 5a–f and Figure 6a–f. This trend was quantitatively validated through the measurement of average fluorescence intensity using confocal laser scanning microscopy, as illustrated in Figure 5g and Figure 6g. These results confirmed that probe P can effectively respond to changes in intracellular levels of Ag^+^ and Hg^2+^ in living cells.

**Figure 7:**
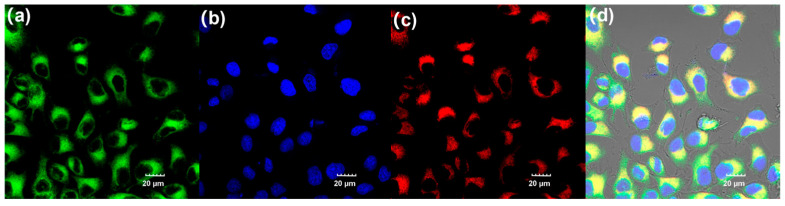
Confocal fluorescence images of Hela cells. (**a**) Cells treated with probe P (10 μM) for 30 min. (**b**) Cells treated with probe P (10 μM) for 30 min, followed by a green live-cell nucleic acid stain. (**c**) Cells treated with probe P (10 μM) for 30 min and then stained with both a green live-cell nucleic acid dye and Mito Tracker Red for 30 min. (**d**) Overlay of panels (**a**–**c**).

As depicted in the blue panel of Figure 7, probe P was predominantly found at the edges of the Mito Tracker Red signal (red panel, Figure 7c). This distribution implied that P was mainly localized in the cytoplasm of living cells rather than within the nucleus (blue panel, Figure 7b). This finding underscored the potential function of P in cytoplasmic activities, suggesting that it could be particularly useful for exploring cytoplasmic environments within biological systems.

## 3. Experimental Section

### 3.1. Reagents and Instruments

All reagents used were of analytical grade and employed without further purification, and the salts used in this work were NaCl, KCl, AgNO_3_, CaCl_2_, MgCl_2_, ZnCl_2_, PbCl_2_, CuCl_2_, HgCl_2_, CdCl_2_, CoCl_2_, NiCl_2_, MnCl_2_, KI, NaNO_3_, NaCl^−^, NaAc, NaClO_4_, NaBr, NaH_2_PO_4_, Na_2_HPO_4_, Na_2_CO_3_, and Na_2_SO_4_. Hela cells were generously provided by professor Yu Fa Biao’s group from Hainan Medical University.

Fluorescence emission spectra were recorded using a Hitachi 4600 spectrofluorometer (Hitachi, Ltd., Chiyoda, Japan), while UV-vis spectra were obtained with a Hitachi U-2910 spectrophotometer (Hitachi, Ltd., Japan). IR spectra were conducted on PerkinElmer Frontier (PerkinElmer Inc., Waltham, MA, America). Mass spectrometry (MS) data were collected on a Thermo TSQ Quantum Access coupled with an Agilent 1100 system (Thermo Fisher Scientific Inc., Waltham, MA, America). Nuclear magnetic resonance (NMR) spectra were measured with a Bruker AV 400 instrument (Bruker Co., Fällanden, Switzerland), with chemical shifts reported in ppm relative to tetramethylsilane (TMS). Cell imaging experiments were conducted using a confocal fluorescence microscope, specifically the Olympus FluoView FV3000 laser scanning microscope (Olympus Co., Tokyo, Japan).

### 3.2. Synthesis

Compounds **1** and **2** were synthesized as our previous work [36].

**Scheme 2:**
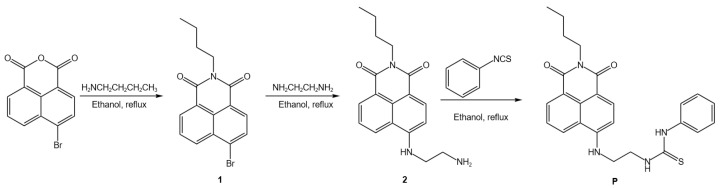
Synthetic route of P.

Synthesis of P (Scheme 2): In a 150 mL three-necked flask, 0.7501 g of compound 2 (2.7 mmol), 0.5 mL of phenyl isothiocyanate (4.0 mmol), and 35 mL of anhydrous ethanol were added. The mixture was stirred and refluxed for 12 h. A large amount of solid was formed after the reaction mixture cooled to room temperature, which was collected by vacuum filtration. The solid was then purified by recrystallization from ethanol, and yellow solid P was obtained. Yields: 76%. IR (KBr, cm^−1^): 3255.9 (N-H), 2954.3 (C=C-H), 2869.5 (C-H), 1678.3 (C=O), 1641.8 (C=S), 1584.7 (C=N), 1537.4 (C=C), 1363.7 (C=C-H), 1243.6 (Ar-H), 772.17 (Ar-H). MS (ESI) *m*/*z*: 444.62 [M-2H]^+^, 446.59 [M]^+^. ^1^H NMR (*d_6_*-DMSO, δ ppm): 11.00 (s, 1H), 9.66 (s, 1H), 8.59 (d, 1H), 8.35 (d, 1H), 8.16 (d, 1H), 7.89 (d, 1H), 7.80 (d, 1H), 7.62 (t, 1H), 7.26 (d, 4H), 7.09 (d, 1H), 6.88 (d, 1H), 4.45 (b, 1H), 3.94 (t, 2H), 3.80 (t, 2H), 3.55 (t, 2H), 1.52 (t, 2H), 1.27 (m, 2H), 0.86 (t, 3H). ^13^C NMR (*d_6_*-DMSO, δ ppm): 181.03, 164.26, 163.43, 151.18, 139.21, 134.61, 131.19, 129.88, 129.42, 129.04, 125.15, 124.85, 124.13, 122.41, 120.65, 108.44, 104.56, 42.73, 30.38, 20.41, 14.33 (Figures S16–S19).

### 3.3. General Spectroscopic Methods

The metal ions, anions, and probe P required for the experiment were dissolved in deionized water and DMSO to prepare 1.0 mM stock solutions. The excitation and emission slit widths were set to 10 nm, with an excitation wavelength of 420 nm.

### 3.4. Reagent Addition

Detection of Ag^+^ in ethanol: In a 5 mL colorimetric tube, 50 μL of P stock solution was added, followed by an appropriate amount of ethanol. Next, the required amount of Ag^+^ stock solution was added, and the total volume was adjusted to 5 mL with ethanol.

Detection of Ag^+^ and Hg^2+^ in ethanol–water solution (*v*:*v*, 9:1, pH 7.0, 20 mM HEPES): In a 5 mL colorimetric tube, 50 μL of P stock solution was added, followed by the necessary amount of Ag^+^ or Hg^2+^ stock solution. The total volume was adjusted to 5 mL using ethanol–water solution (*v*:*v*, 9:1, pH 7.0, 20 mM HEPES).

### 3.5. Cell Incubation and Imaging

Hela cells cultured on coverslips were rinsed with phosphate-buffered saline (PBS) and subsequently incubated with 10 μM of probe P for 30 min at 37 °C. Following this, the cells were washed three times with PBS. The cells were then incubated with 10 μM of Ag^+^ (diluted in PBS) for 30 min at 37 °C and washed three additional times with PBS. Intracellular imaging of Ag^+^ in Hela cells was performed using a confocal fluorescence microscope (Olympus FluoView Fv3000, Olympus Co., Japan). Other cellular experimental procedures were carried out as previously described.

## 4. Conclusions

In summary, we developed a novel fluorescent probe P, based on naphthalimide, specifically designed for the selective detection of Ag^+^ and Hg^2+^. This probe exhibited a significant Stokes shift of 107 nm and maintained high stability across a broad pH range. Upon the addition of Ag^+^ or Hg^2+^ to a solution of P in either ethanol or an ethanol/water mixture (9:1, *v*:*v*), we observed a pronounced decrease in fluorescence emission at 537 nm, accompanied by the emergence of dual absorption wavelengths and a color change from yellow to colorless. The introduction of other common metal ions did not produce any significant alterations in the spectra or color. Additionally, the probe demonstrated a rapid response to Ag^+^ or Hg^2+^ within just 5 min. Meanwhile, cell imaging indicated the potential for sensing these ions in living cells.

## Data Availability

Data will be made available on request.

## Supplementary Materials

The following supporting information can be downloaded at: https://www.mdpi.com/article/10.3390/molecules29215196/s1, Figure S1 (a) Fluorescence response of P (10 µM) to Ag^+^ (10 µM) or to the mixture of individual metal ions (50 µM) with Ag^+^ (10 µM) in ethanol; (b) Fluorescence response of P (10 µM) to Ag^+^ (10 µM) or to the mixture of individual metal ions (50 µM) with Ag^+^ (10 µM) in ethanol-water solution (*v*:*v*, 9:1, pH 7.0, 20 mM HEPES); (c) Fluorescence response of P (10 µM) to Ag^+^ (10 µM) or to the mixture of individual metal ions (50 µM) with Hg^2+^ (10 µM) in ethanol-water solution (*v*:*v*, 9:1, pH 7.0, 20 mM HEPES); Figure S2 The reversibility of the P-Ag^+^ system was investigated in ethanol-water solution (*v*:*v*, 9:1, pH7, 20 mM HEPES): a. P (10 μM); b. P (10 μM) + Ag^+^ (10 μM); c. P (10 μM) + Ag^+^ (10 μM) + Cl^−^ (10 μM); d. P (10 μM) + Ag^+^ (10 μM) + Cl^−^ (100 μM); e. P (10 μM) + Ag^+^ (10 μM) + Cl^−^ (10 μM) + Ag^+^ (100 μM); Figure S3 The reversibility of the P-Hg^2+^ system was investigated in ethanol-water solution (*v*:*v*, 9:1, pH7, 20 mM HEPES): a. P (10 μM); b. P (10 μM) + Hg^2+^ (10 μM); c. P (10 μM) + Hg^2+^ (10 μM) + S^2−^ (10 μM); d. P (10 μM) + Hg^2+^ (10 μM) + S^2−^ (100 μM); e. P (10 μM) + Hg^2+^ (10 μM) + S^2−^ (10 μM) + Hg^2+^ (100 μM); Figure S4 The effect of different solvents on the probe’s spectrum. (a) Fluorescence; (b) UV-vis; Figure S5 Effect of different water contents on the fluorescence spectra of probe P (10 μM) and P (10 μM)-Ag^+^ (100 μM), P (10 μM)-Hg^2+^ (100 μM); Figure S6 Effect of different pH values on the fluorescence spectra of probe P (10 μM) and P (10 μM)-Ag^+^ (100 μM) or Hg^2+^ (100 μM) in ethanol-water solution (*v*:*v*, 9:1); Figure S7 Effect of equilibrium time of probe P (10 μM) and Ag^+^ (100 μM) on fluorescence intensity in ethanol-water solution (*v*:*v*, 9:1, pH 7.0, 20 mM HEPES); Figure S8 Effect of equilibrium time of probe P (10 μM) and Hg^2+^ (100 μM) on fluorescence intensity in ethanol-water solution (*v*:*v*, 9:1, pH 7.0, 20 mM HEPES); Figure S9 Photostability tests of P (10 μM) and P (10 μM) plus Ag^+^ (100 μM) in ethanol; P (10 μM) and P (10 μM) plus Ag^+^ (100 μM), P (10 μM) plus Hg^2+^ (100 μM) in ethanol-water solution (*v*:*v*, 9:1, pH 7.0, 20 mM HEPES); Figure S10 ^1^H NMR spectra of P-Ag^+^; Figure S11 ^1^H NMR spectra of P-Hg^2+^; Figure S12 ESI-MS spectra of P-Ag^+^; Figure S13 ESI-MS spectra of P-Hg^2+^; Figure S14 Benesi-Hildebrand plot of P, assuming 1:1 stoichiometry for association between P and Ag^+^; Figure S15 Benesi-Hildebrand plot of P, assuming 1:1 stoichiometry for association between P and Hg^2+^; Figure S16 FT-IR of compounds; Figure S17 ^1^H NMR of P; Figure S18 ^13^C NMR of P; Figure S19 ESI-MS of P; Table S1 Comparison of performances of various fluorescence methods for determination of Ag^+^/Hg^2+^.
