# Supplementary material for: Detection of Silver and Mercury Ions Using Naphthalimide-Based Fluorescent Probe
Source: Molecules. 2024 Nov 2;29(21):5196. doi: 10.3390/molecules29215196 (PMC11547768; doi:10.3390/molecules29215196)
Supplement: Supplementary file 1 [file molecules-29-05196-s001.zip › molecules-3268807-supplementary.pdf]

Supporting Information

Detection of Silver and Mercury Ions Using Naphthalimide-Based Fluorescent Probes

Chunwei Yu †, Xiangxiang Li †, Mei Yang, Yinghao Xie and Jun Zhang*

NHC Key Laboratory of Control of Tropical Diseases, School of Tropical Medicine, Hainan Medical University, Haikou 571199, China; cwyu@muhn.edu.cn (C.Y.); lxx@hainmc.edu.cn (X.L.); yang24364@hainmc.edu.cn (M.Y.); sos3126444394@gmail.com (Y.X.)

* Correspondence: hy0211045@muhn.edu.cn; Tel.: +86-898-66965257; Fax: +86-898-66989173

† These authors contributed equally to this work.

Contents

Figure S1 a) Fluorescence response of P (10 μM) to Ag^+ (10 μM) or to the mixture of individual metal ions (50 μM) with Ag^+ (10 μM) in ethanol; b) Fluorescence response of P (10 μM) to Ag^+ (10 μM) or to the mixture of individual metal ions (50 μM) with Ag^+ (10 μM) in ethanol-water solution (v:v, 9:1, pH 7.0, 20 mM HEPES); c) Fluorescence response of P (10 μM) to Ag^+ (10 μM) or to the mixture of individual metal ions (50 μM) with Hg^{2+} (10 μM) in ethanol-water solution (v:v, 9:1, pH 7.0, 20 mM HEPES).

Figure S2 The reversibility of the P-Ag^+ system was investigated in ethanol-water solution (v:v, 9:1, pH7, 20 mM HEPES): a. P (10 μM); b. P (10 μM) + Ag^+ (10 μM); c. P (10 μM) + Ag^+ (10 μM) + Cl^- (10 μM); d. P (10 μM) + Ag^+ (10 μM) + Cl^- (100 μM); e. P (10 μM) + Ag^+ (10 μM) + Cl^- (10 μM) + Ag^+ (100 μM).

Figure S3 The reversibility of the P-Hg^{2+} system was investigated in ethanol-water solution (v:v, 9:1, pH7, 20 mM HEPES): a. P (10 μM); b. P (10 μM) + Hg^{2+} (10 μM); c. P (10 μM) + Hg^{2+} (10 μM) + S^{2-} (10 μM); d. P (10 μM) + Hg^{2+} (10 μM) + S^{2-} (100 μM); e. P (10 μM) + Hg^{2+} (10 μM) + S^{2-} (10 μM) + Hg^{2+} (100 μM).

Figure S4 The effect of different solvents on the probe's spectrum. a) Fluorescence; b) UV-vis.

Figure S5 Effect of different water contents on the fluorescence spectra of probe P (10 μM) and P (10 μM)-Ag^+ (100 μM), P (10 μM)-Hg^{2+} (100 μM).

Figure S6 Effect of different pH values on the fluorescence spectra of probe P (10 μM) and P (10 μM)-Ag^+ (100 μM) or Hg^{2+} (100 μM) in ethanol-water solution (v:v, 9:1).

Figure S7 Effect of equilibrium time of probe P (10 μM) and Ag^+ (100 μM) on fluorescence intensity in ethanol-water solution (v:v, 9:1, pH 7.0, 20 mM HEPES).

Figure S8 Effect of equilibrium time of probe P (10 μM) and Hg^{2+} (100 μM) on fluorescence intensity in ethanol-water solution (v:v, 9:1, pH 7.0, 20 mM HEPES).

Figure S9 Photostability tests of P (10 μM) and P (10 μM) plus Ag^+ (100 μM) in ethanol; P (10 μM) and P (10 μM) plus Ag^+ (100 μM), P (10 μM) plus Hg^{2+} (100 μM) in ethanol-water solution (v:v, 9:1, pH 7.0, 20 mM HEPES).

Figure S10 ^1H NMR spectra of P-Ag^+.

Figure S11 ^1H NMR spectra of P-Hg^{2+}.

Figure S12 ESI-MS spectra of P-Ag^+.

Figure S13 ESI-MS spectra of P-Hg^{2+}.

Figure S14 Benesi-Hildebrand plot of P, assuming 1:1 stoichiometry for association between P and Ag^+.

Figure S15 Benesi-Hildebrand plot of P, assuming 1:1 stoichiometry for association between P and Hg^{2+}.

Figure S16 FT-IR of compounds.

Figure S17 ^1H NMR of P.

Figure S18 ^{13}C NMR of P.

Figure S19 ESI-MS of P.

Table S1 Comparison of performances of various fluorescence methods for determination of Ag^+/Hg^{2+}.

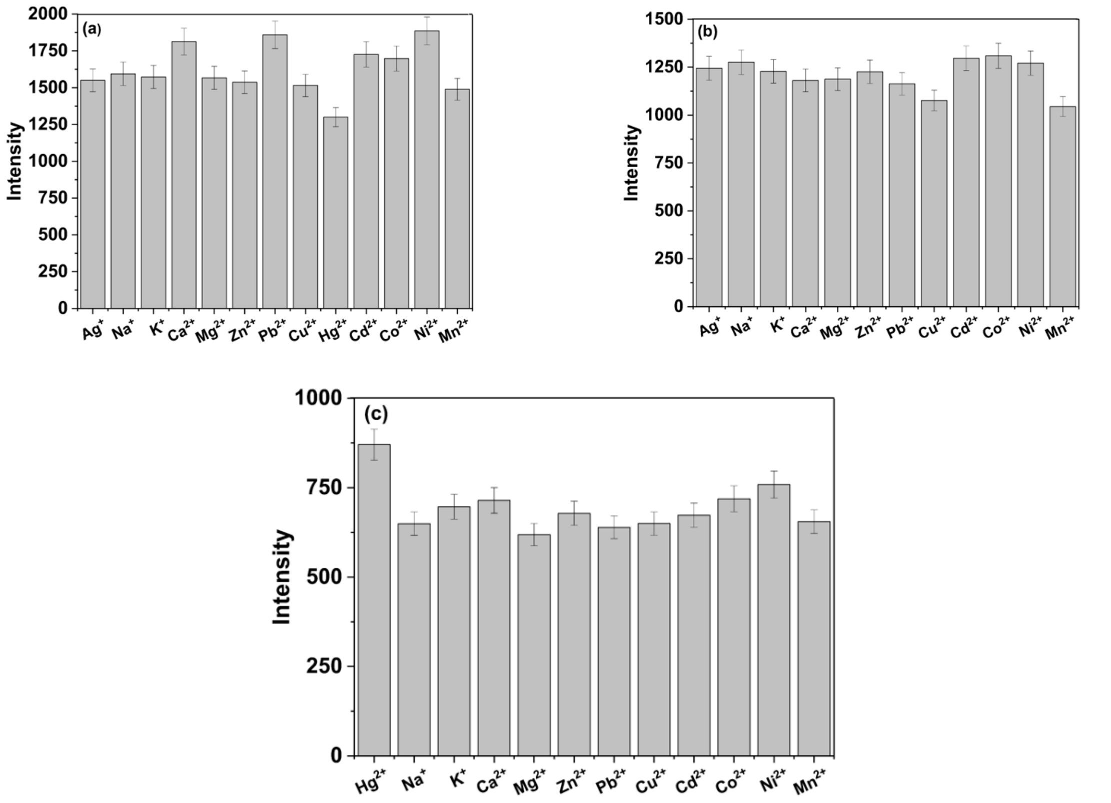

Figure S1 (a) Fluorescence response of P (10 μM) to Ag^{+} (10 μM) or to the mixture of individual metal ions (50 μM) with Ag^{+} (10 μM) in ethanol; (b) Fluorescence response of P (10 μM) to Ag^{+} (10 μM) or to the mixture of individual metal ions (50 μM) with Ag^{+} (10 μM) in ethanol-water solution (v:v, 9:1, pH 7.0, 20 mM HEPES); (c) Fluorescence response of P (10 μM) to Ag^{+} (10 μM) or to the mixture of individual metal ions (50 μM) with Hg^{2+} (10 μM) in ethanol-water solution (v:v, 9:1, pH 7.0, 20 mM HEPES).

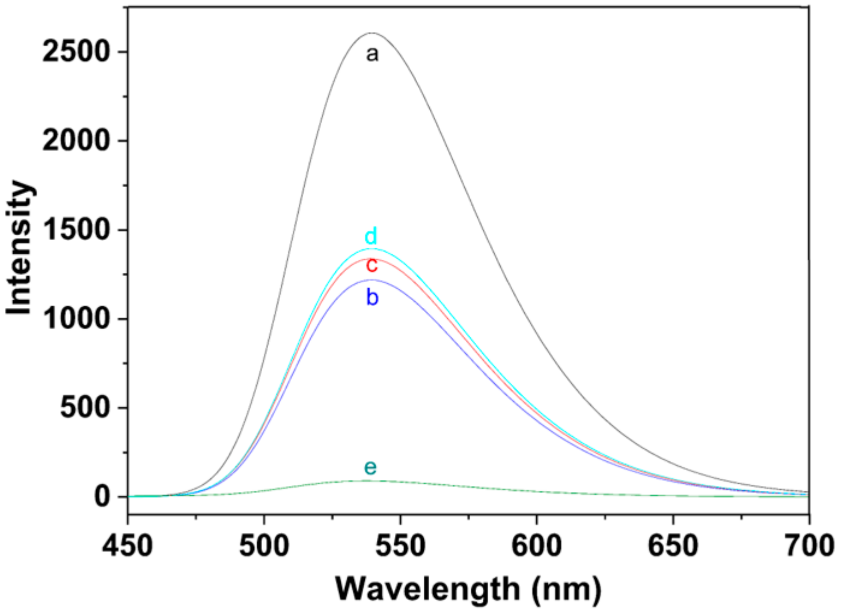

Figure S2 The reversibility of the P-Ag^+ system was investigated in ethanol-water solution (v:v, 9:1, pH7, 20 mM HEPES): a. P (10 μM); b. P (10 μM) + Ag^+ (10 μM); c. P (10 μM) + Ag^+ (10 μM) + Cl^- (10 μM); d. P (10 μM) + Ag^+ (10 μM) + Cl^- (100 μM); e. P (10 μM) + Ag^+ (10 μM) + Cl^- (10 μM) + Ag^+ (100 μM).

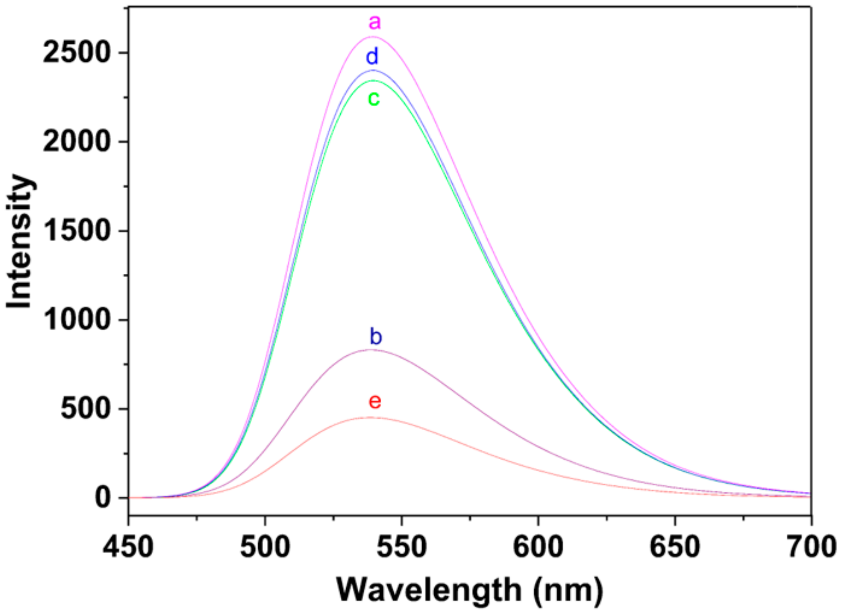

Figure S3 The reversibility of the P-Hg^{2+} system was investigated in ethanol-water solution (v:v, 9:1, pH7, 20 mM HEPES): a. P (10 μM); b. P (10 μM) + Hg^{2+} (10 μM); c. P (10 μM) + Hg^{2+} (10 μM) + S^{2-} (10 μM); d. P (10 μM) + Hg^{2+} (10 μM) + S^{2-} (100 μM); e. P (10 μM) + Hg^{2+} (10 μM) + S^{2-} (10 μM) + Hg^{2+} (100 μM).

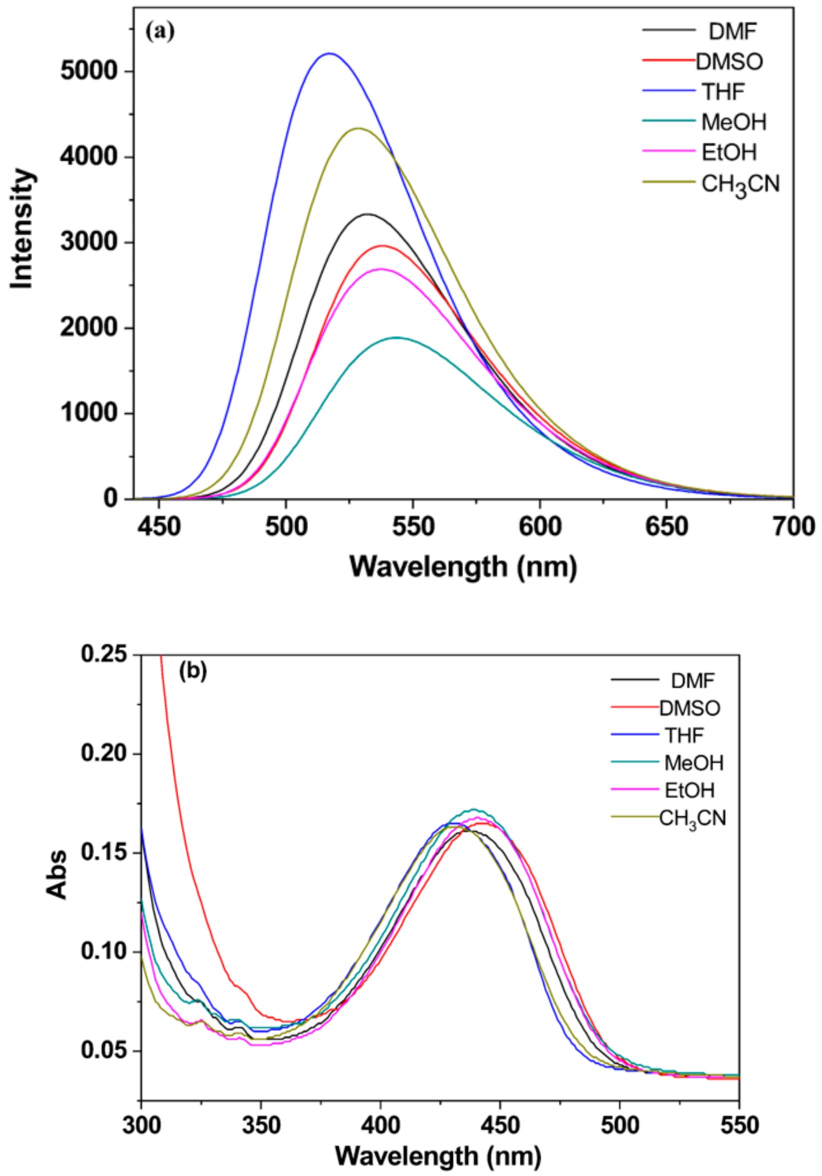

Figure S4 The effect of different solvents on the probe's spectrum a) Fluorescence; b) UV-vis.

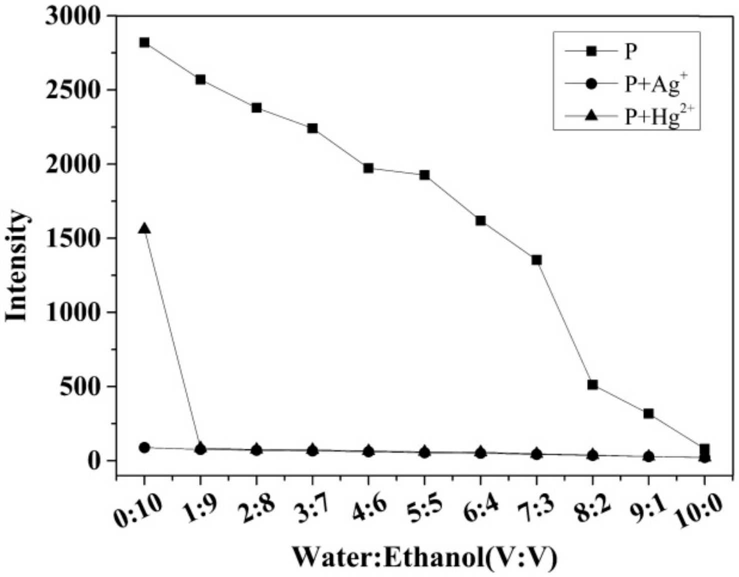

Figure S5 Effect of different water contents on the fluorescence spectra of probe P (10 μM) and P (10 μM)-Ag^+ (100 μM), P (10 μM)-Hg^{2+} (100 μM).

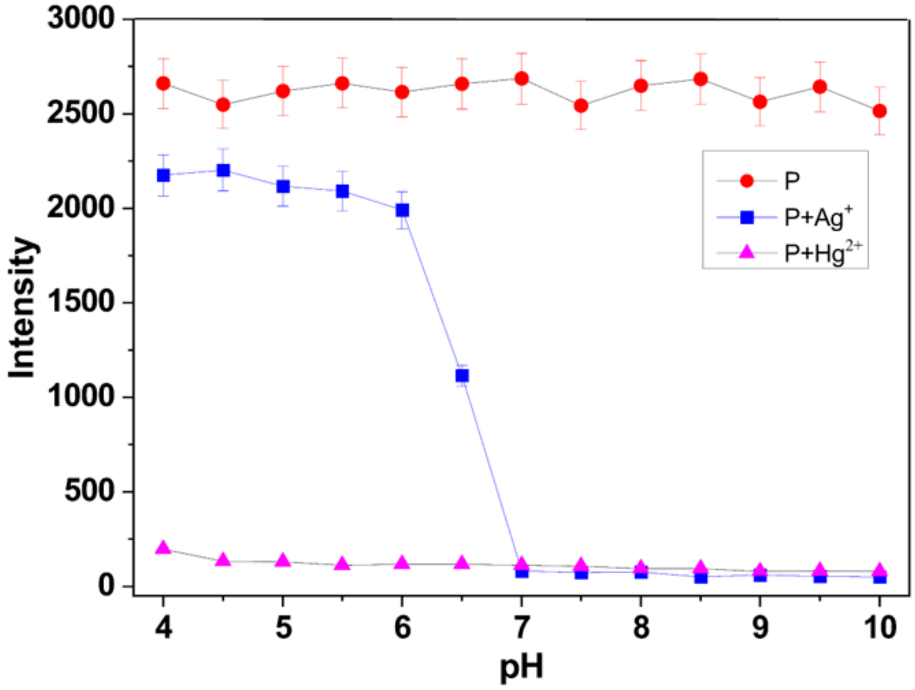

Figure S6 Effect of different pH values on the fluorescence spectra of probe P (10 μM) and P (10 μM)-Ag^{+} (100 μM) or Hg^{2+} (100 μM) in ethanol-water solution (v:v, 9:1).

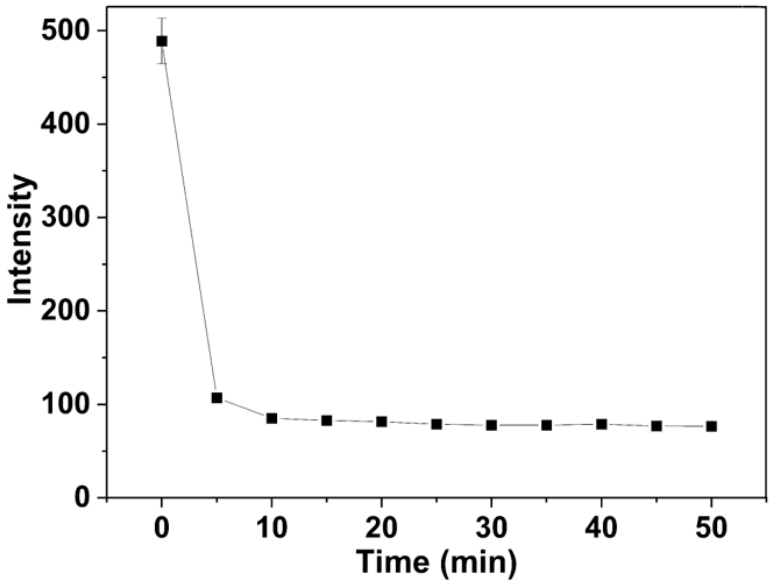

Figure S7 Effect of equilibrium time of probe P (10 μM) and Ag^+ (100 μM) on fluorescence intensity in ethanol-water solution (v:v, 9:1, pH 7.0, 20 mM HEPES).

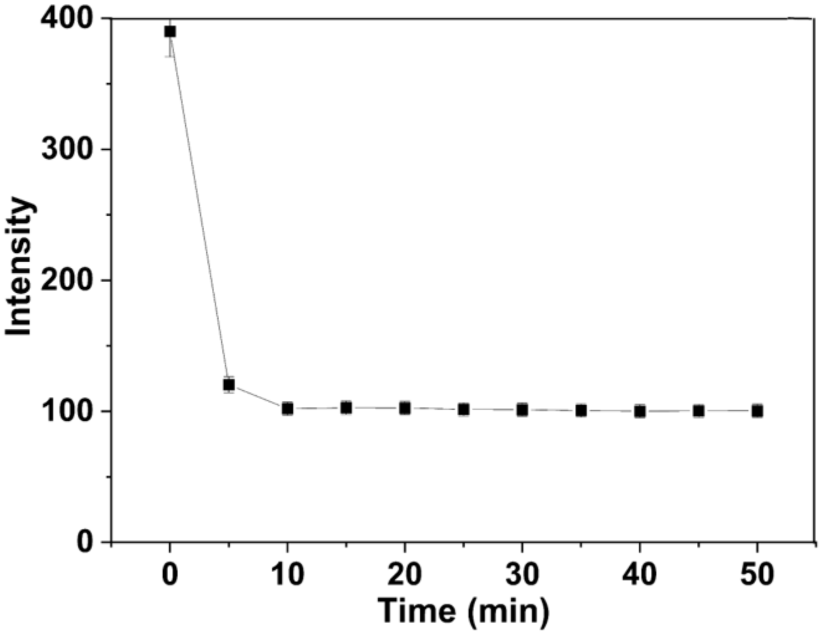

Figure S8 Effect of equilibrium time of probe P (10 μM) and Hg^{2+} (100 μM) on fluorescence intensity in ethanol-water solution (v:v, 9:1, pH 7.0, 20 mM HEPES).

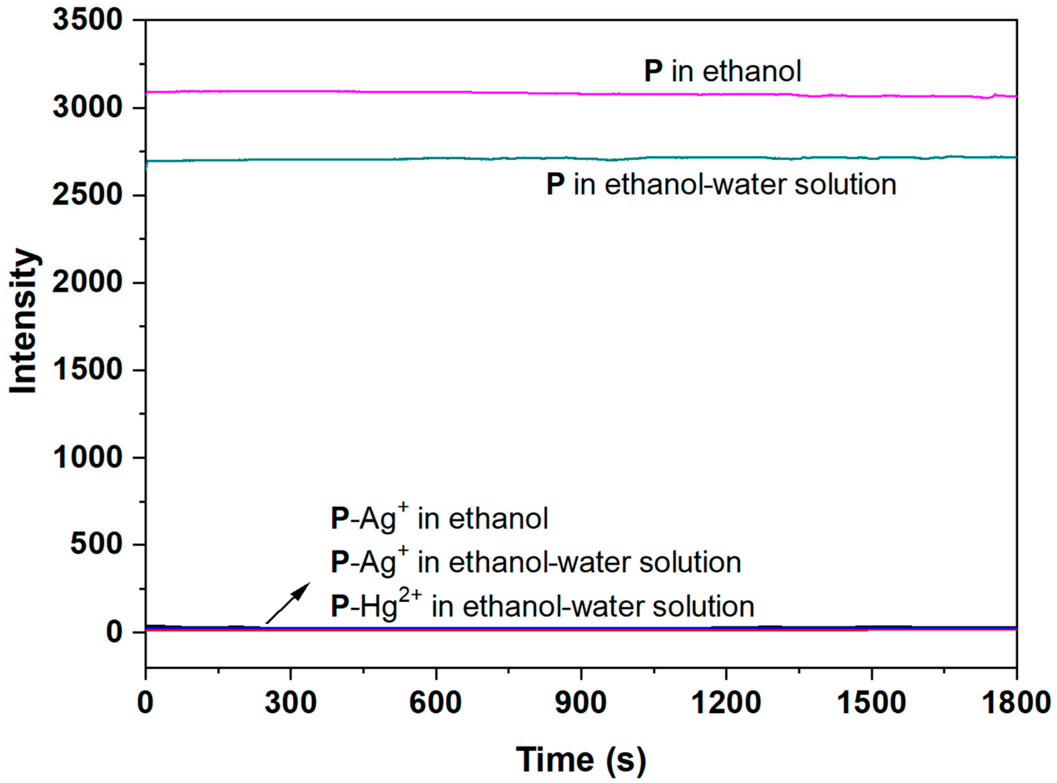

Figure S9 Photostability tests of P (10 μM) and P (10 μM) plus Ag^{+} (100 μM) in ethanol; P (10 μM) and P (10 μM) plus Ag^{+} (100 μM), P (10 μM) plus Hg^{2+} (100 μM) in ethanol-water solution (v:v, 9:1, pH 7.0, 20 mM HEPES).

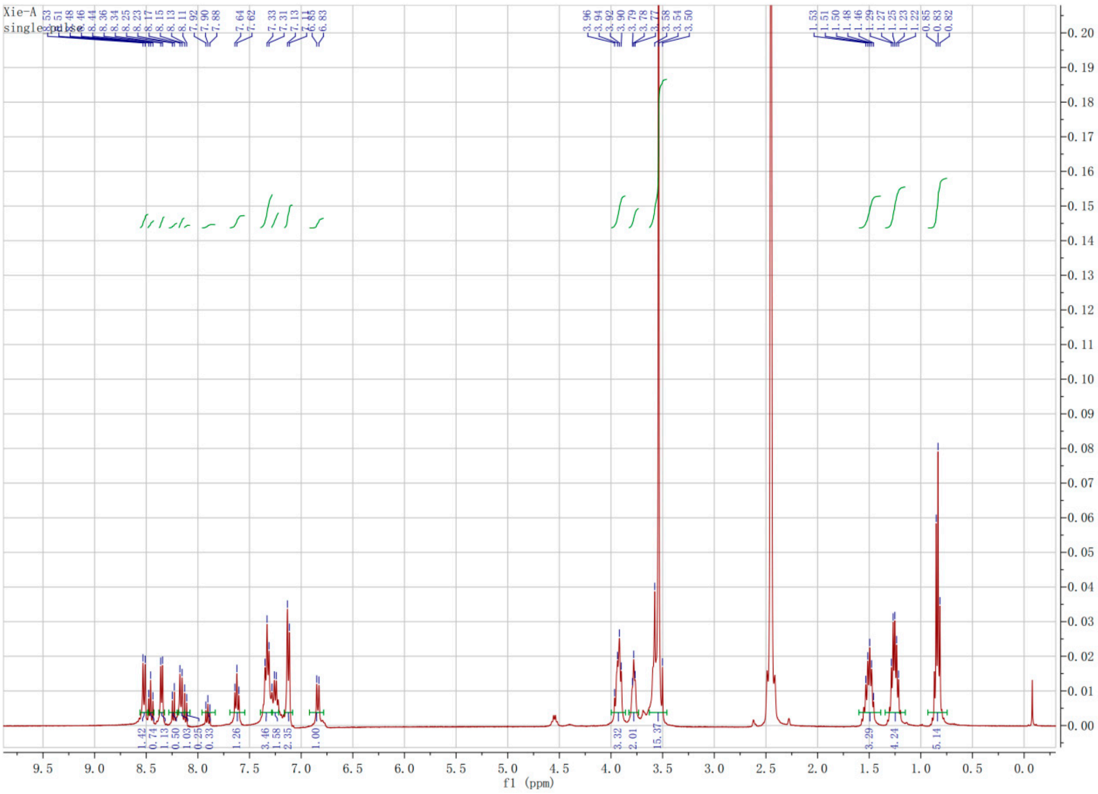

Figure S10 ^{1}H NMR spectra of P-Ag^{+}.

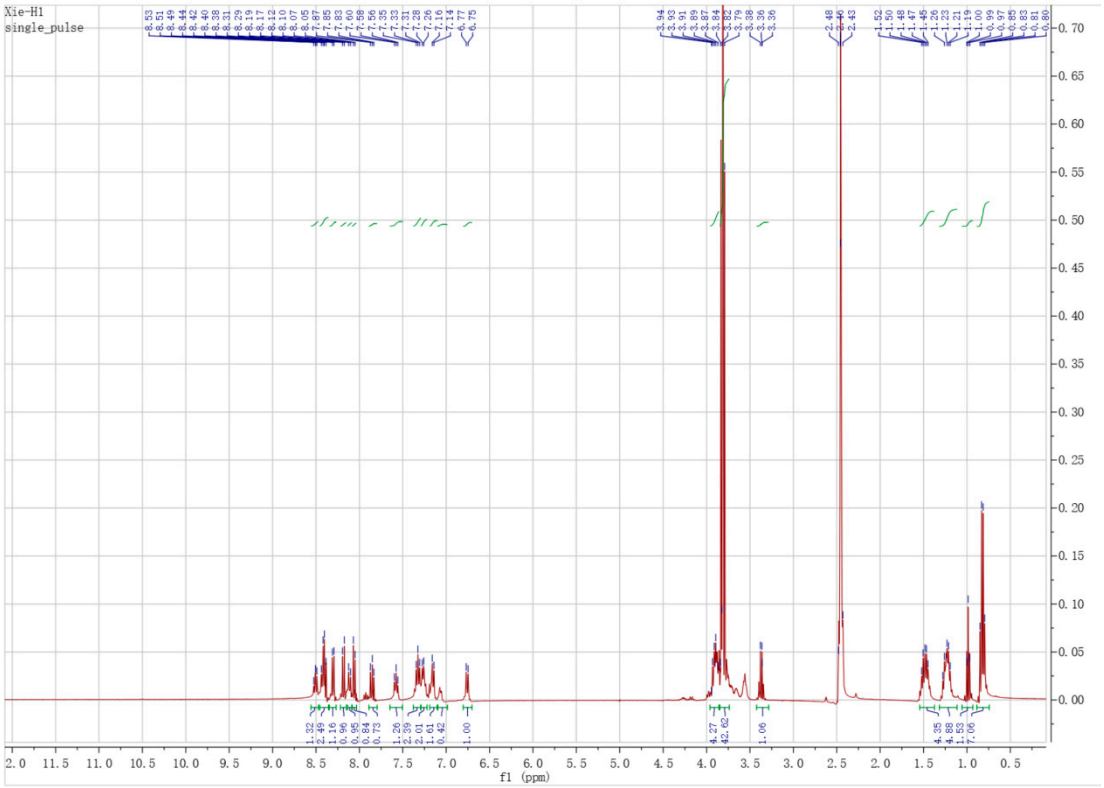

Figure S11 ^{1}H NMR spectra of P-Hg^{2+}.

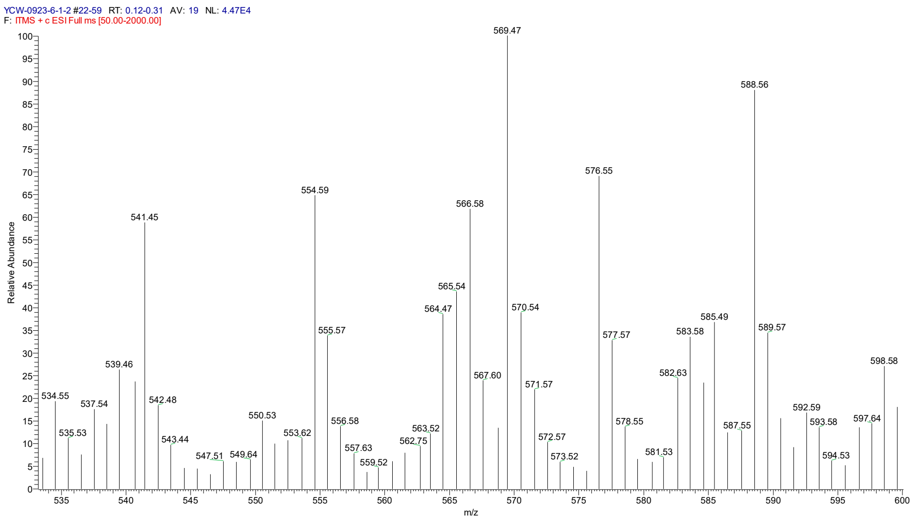

Figure S12 ESI-MS spectra of P-Ag^{+}.

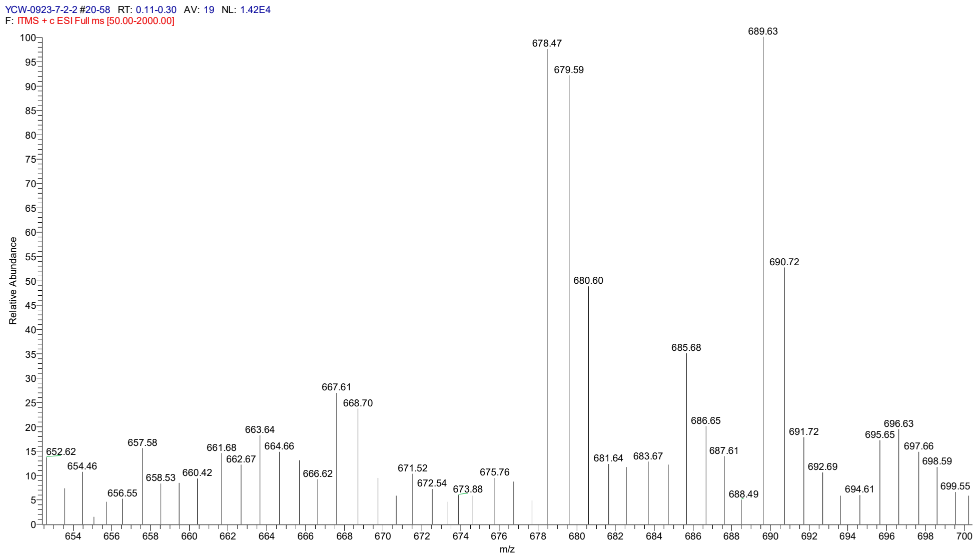

Figure S13 ESI-MS spectra of P-Hg^{2+}.

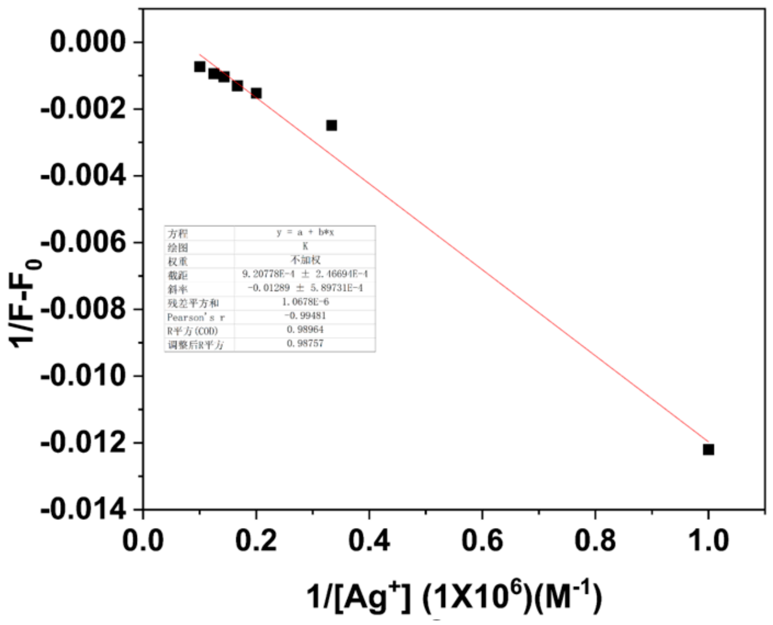

Figure S14 Benesi-Hildebrand plot of P, assuming 1:1 stoichiometry for association between P and Ag^{+}.

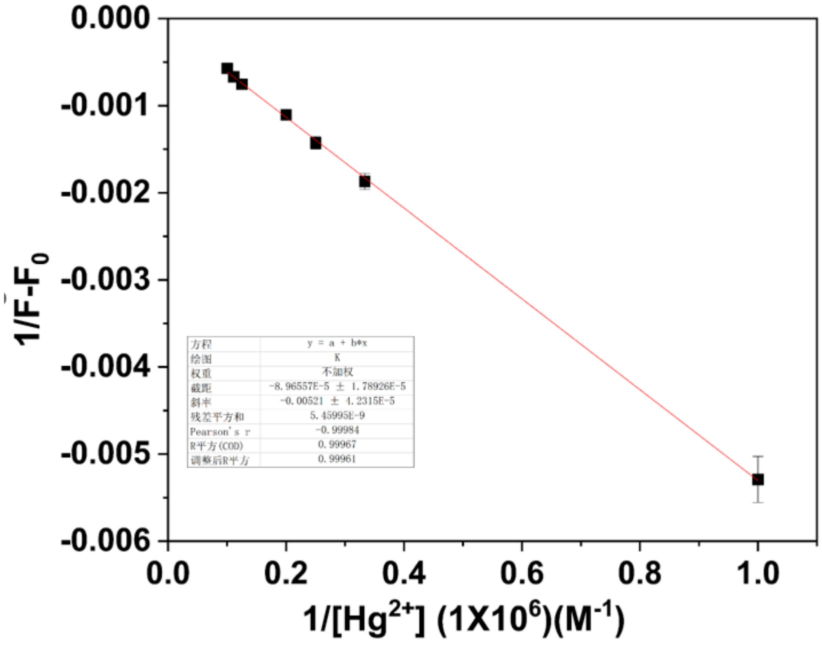

Figure S15 Benesi-Hildebrand plot of P, assuming 1:1 stoichiometry for association between P and Hg^{2+}.

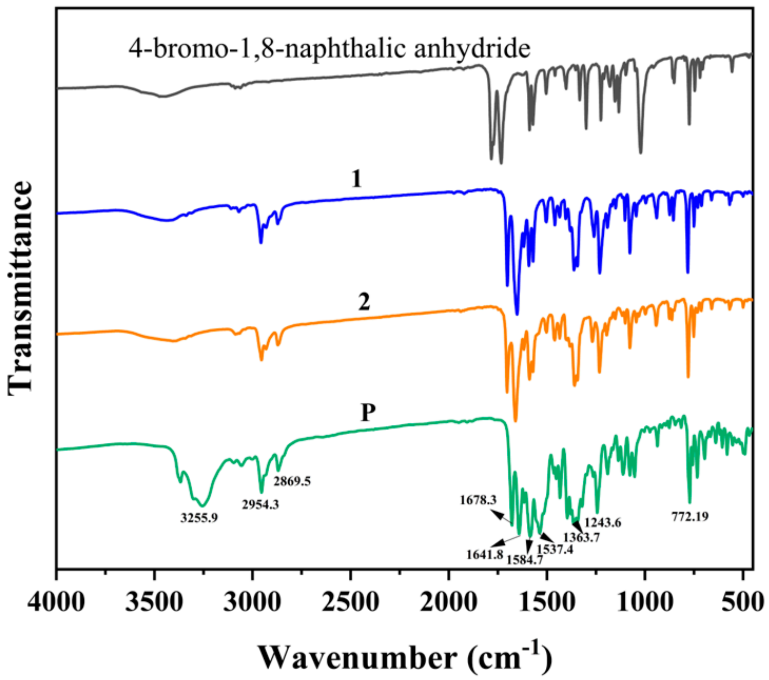

Figure S16 FT-IR of compounds.

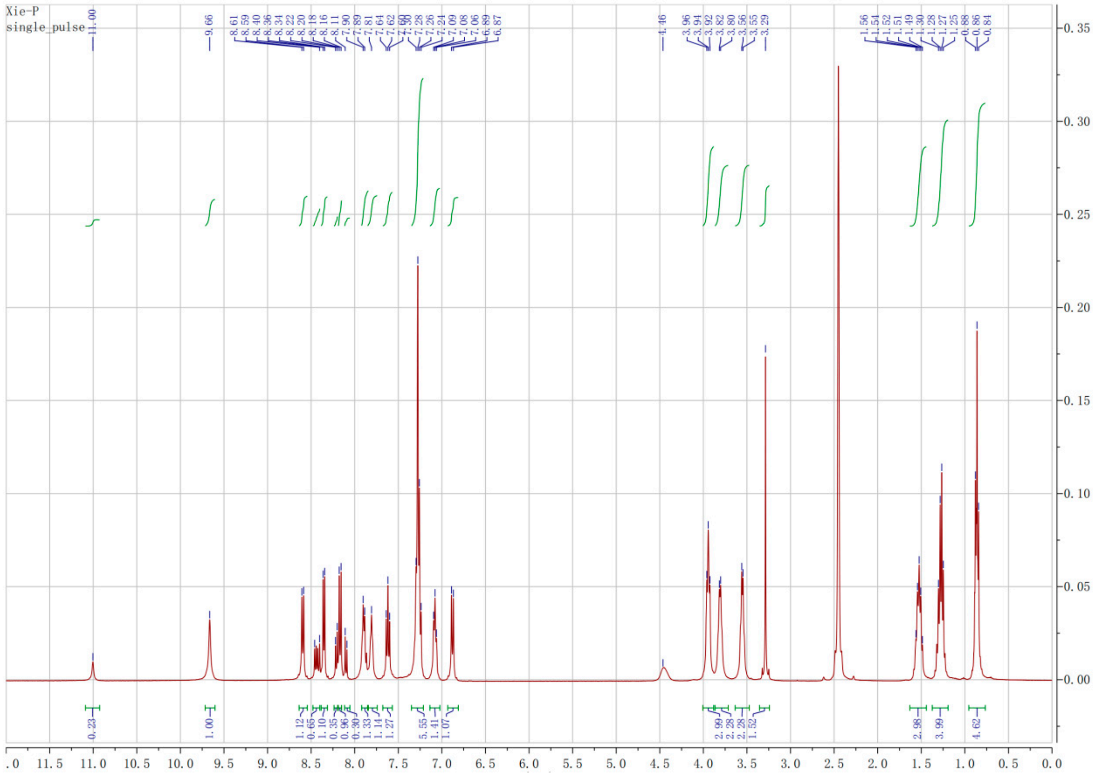

Figure S17 ^{1}H NMR of P.

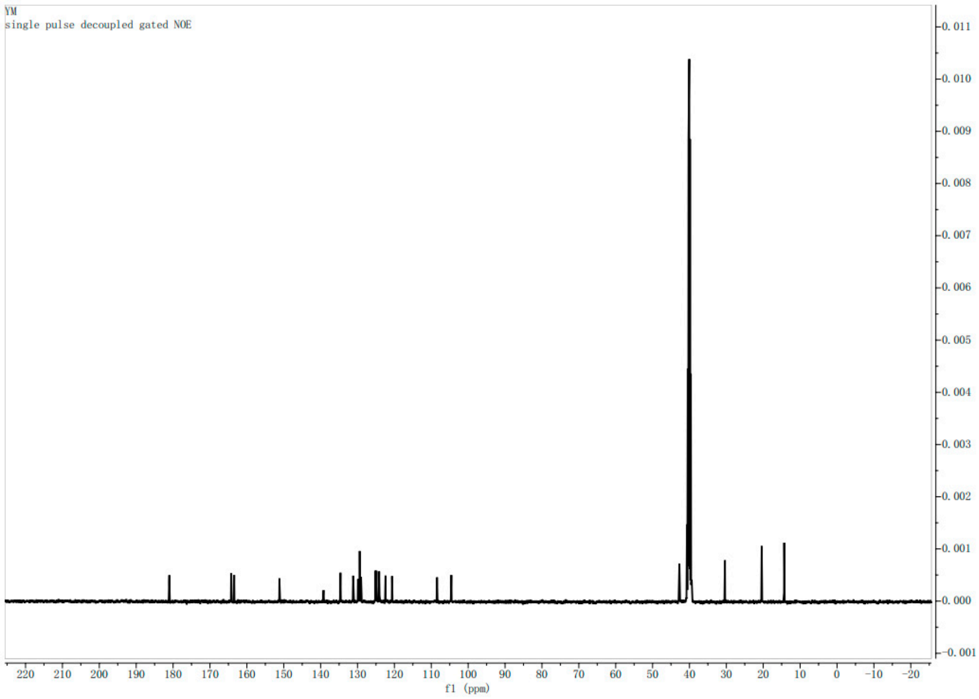

Figure S18 ^{13}C NMR of P.

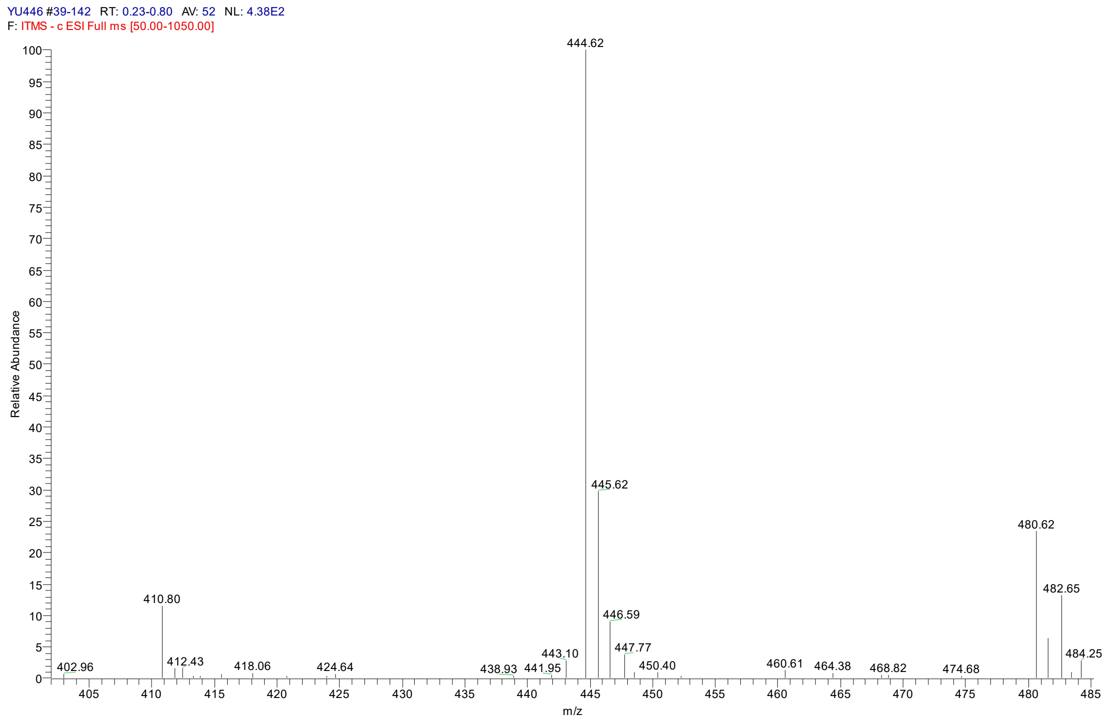

Figure S19 ESI-MS of P.

Table S1 Comparison of performances of various fluorescence methods for determination of Ag^+/Hg^{2+}.

Detection mode	Probe	Selectivity	Linear range, μM	LOD, μM	Detection media	Response time, min	Applications	Refs
Turn-off/ UV-visible	BODIPY derivative	Ag^+, Hg^{2+}	0-10, 0-14	0.25	DMF/Buffer (8:2, pH 7.0)	NM	Water samples	[28]
Turn-off/ UV-visible	Naphthalimide derivative	Ag^+, Hg^{2+}	0-24, 0-17	1.20, 0.83	MeCN/H_2O (4:1, v:v)	15	Water samples and cell imaging	[29]
Turn-off/ UV-visible	Isonicotinohydrazide derivative	Ag^+, Hg^{2+}	2-20. 2-20	1.1, 0.72	EtOH/H_2O (9:1, v:v)	1-3	NM	[30]
Turn-off/ UV-visible	Bianthracene derivative	Ag^+, Hg^{2+}	30-50, 0.5-20	19.9, 0.662	DMF/H_2O (9:1, v:v, pH 3.0)	NM	NM	[31]
Turn-on/ UV-visible	Coumarin derivative	Ag^+, Hg^{2+}	2-20, 0.02-0.20	8.8, 0.83	EtOH/H_2O (1:1, v:v)	10, 3	Cell imaging	[32]
Turn-on	Rhodol derivative	Ag^+, Hg^{2+}	0-5, 0-4	0.45, 0.27	THF/Buffer (4:6, v:v, pH 7.4)	NM	Cell imaging	[33]
Turn-off/ turn-on/ UV-visible	Cinnamaldehyde derivative	Ag^+, Hg^{2+}	NM	5, 2	CH_3CN/Buffer (1:1, v:v, 10 mM, pH 7.4)	30	Water samples	[34]
Turn-on/ UV-visible	Pyridine derivative	Ag^+, Hg^{2+}	50-200, 0.1-18	3.67, 0.00069	CH_3OH/H_2O (1:4, v:v)	NM	Cell imaging	[35]
Turn-off/ UV-visible	Naphthalimide derivative	Ag^+, Hg^{2+}	1-10, 1-10	0.33	EtOH/H_2O, (9:1, v:v, 20 mM Buffer, pH 7.0)	5	Cell imaging	This work
